# Effects of a somatic dance course on body image, embodied self-awareness, and disordered eating risk in female university students: a quasi-experimental study

**DOI:** 10.3389/fpsyg.2026.1933772

**Published:** 2026-10-19

**Authors:** Jiage Yin

**Affiliations:** Normal College, Xuzhou University of Technology, Xuzhou, China

**Keywords:** body image, disordered eating, embodied self-awareness, embodiment, female university students, positive body image, quasi-experimental study, somatic dance

## Abstract

**Introduction:**

Body image concerns and disordered eating risk are common among female university students, and contemporary frameworks increasingly emphasize positive body image and embodiment. This quasi-experimental, pre-post controlled study examined whether an 8-week somatic-oriented improvisational dance course, delivered as a non-competitive university body education elective and compared with regular university physical education electives, was associated with changes in body appreciation, embodied self-awareness, disordered eating risk, functionality appreciation, and body surveillance.

**Methods:**

A total of 128 female undergraduates were allocated to the somatic dance course or comparison condition (64 per group). Post-intervention questionnaires were completed by 115 participants, and the main attention-check-passing analysis sample comprised 103 participants. Group differences were estimated using ANCOVA models adjusted for baseline scores, with baseline BMI as an additional covariate.

**Results:**

Relative to regular university physical education electives, the somatic dance course was associated with greater post-intervention body appreciation (BAS-2), embodied self-awareness (SBC), and functionality appreciation (FAS) and lower body surveillance (OBCS-BS). Adjusted group differences were statistically significant for BAS-2, SBC, FAS, and OBCS-BS, whereas the group difference in disordered eating risk (EAT-26) was not statistically significant. Exploratory change-score mediation analyses did not support an indirect pathway through change in embodied self-awareness.

**Conclusion:**

The 8-week somatic dance course was associated with short-term group differences in positive body-image and embodiment-related outcomes among female university students. The group difference in disordered eating risk was not statistically significant, and mediation pathways were not supported, indicating the need for rigorous randomized studies with longer follow-up.

## 1 Introduction

### 1.1 Body image concerns and disordered eating risk in female university students

Body image concerns are among the most common psychological difficulties experienced by young women, and the university years represent a developmental window of heightened vulnerability. During this period, identity consolidation, intensified appearance comparison, changing social environments, and shifts in eating and activity routines converge in ways that can elevate disordered eating risk. Meta-analytic evidence indicates that body dissatisfaction, dieting, and negative affect are robust antecedents of eating pathology, operating across diverse populations and developmental stages (Stice, 2002). In women specifically, distinct facets of body image prospectively predict disordered eating, and configurations of body shame, body appreciation, and body mass index differentiate patterns of dietary restraint and exercise (Raspovic et al., 2023; Anderson and Linardon, 2024).

These risk processes are increasingly shaped by the contemporary sociocultural environment. Weight stigma is associated with disordered eating cognitions and behaviors (Levinson et al., 2024), and a growing body of systematic reviews and meta-analyses links social-media use, eating-related online content, and diet- and fitness-monitoring applications to poorer body image and elevated disordered eating symptomatology, particularly among young women (Wu et al., 2024; Anderberg et al., 2025; Zhang et al., 2025). Recent work further suggests that physical activity and body image can operate as protective or risk pathways linking social-media use to disordered eating in young adults (Yu et al., 2026). Because the great majority of affected individuals never reach a clinical threshold yet still experience substantial distress, there is broad consensus that prevention and early, non-clinical support are needed alongside formal treatment. The present study is positioned within this preventive, supportive tradition: it targets a non-clinical sample of female university students and aims to strengthen protective body-image resources rather than to treat diagnosed eating disorders.

### 1.2 From negative body image to positive body image

Body image research has expanded beyond the reduction of body dissatisfaction toward the cultivation of positive body image, a multifaceted construct encompassing acceptance of, respect for, and care for the body irrespective of appearance (Tylka and Wood-Barcalow, 2015b). A central, well-operationalized indicator of this construct is body appreciation, captured by the Body Appreciation Scale-2 (BAS-2; Tylka and Wood-Barcalow, 2015a). A systematic review and meta-analysis has shown that body appreciation is consistently associated with better psychological functioning and with lower levels of disordered eating (Linardon et al., 2022), and prospective evidence indicates that positive body image protects against the onset of core eating-disorder symptoms and predicts decreases in eating pathology, partly through intuitive eating (Linardon, 2021; Messer et al., 2022). Comparable associations among body appreciation, gratitude, and intuitive eating have been reported in Asian women (Yamamiya et al., 2021).

A complementary facet of positive body image is functionality appreciation—valuing and being grateful for what the body is able to do, across sensory, physical, and self-care domains—assessed with the Functionality Appreciation Scale (FAS; Alleva et al., 2017) and reviewed conceptually within the broader literature on body functionality (Alleva and Tylka, 2021). Functionality appreciation has identifiable prospective predictors and is linked to favorable body-image and eating outcomes, including among individuals with eating disorders (Liu et al., 2025; Ramesh et al., 2026). Because positive body image is broader than appearance evaluation, sustainable change is thought to require improvements across several of its facets rather than appearance satisfaction alone (Tylka and Wood-Barcalow, 2015b; Linardon et al., 2022). The present study therefore measured both body appreciation and functionality appreciation, anticipating that a body-oriented program would shift evaluation from how the body looks toward what it can do.

### 1.3 Embodiment, objectification, and disordered eating risk

Understanding why body-oriented programs might work requires moving beyond whether women like their appearance to whether the body is experienced as a site of agency, sensation, connection, and self-care. The developmental theory of embodiment frames this lived quality as the experience of embodiment, which spans body connection and comfort, agency and functionality, attuned self-care, and freedom from objectification (Piran, 2016). A closely related construct is body connection—the felt sense of awareness of and connection to internal bodily states—which can be assessed with the Scale of Body Connection (Price and Thompson, 2007) and provided the basis for the embodied self-awareness measure used here. Internal body orientation of this kind is associated with body appreciation and adaptive eating (Modica, 2021).

Objectification theory offers a complementary account of risk. It proposes that women are socialized to internalize an observer’s perspective on their own bodies, leading to habitual body surveillance—the chronic monitoring of outward appearance—indexed by the Body Surveillance subscale of the Objectified Body Consciousness Scale (OBCS; McKinley and Hyde, 1996). Self-objectification and body surveillance are linked, via body shame, to disordered eating and related difficulties (Fredrickson and Roberts, 1997; Noll and Fredrickson, 1998). Recent meta-analytic evidence supports these links from complementary angles: Saunders et al. (2024) quantified the relations among body shame, self-objectification, and body dissatisfaction; Guo et al. (2025) showed that self-objectification remains gendered across measures and societal contexts; and Wang et al. (2025) synthesized evidence linking social media use with self-objectification. In Chinese women specifically, self-objectification and body comparison have been implicated in disordered eating (Xu and Chen, 2025). Conversely, functionality appreciation is associated with lower self-objectification and better well-being (Golmohamadi et al., 2025). Together, these literatures suggest that interventions which strengthen body connection and embodied self-awareness and reduce body surveillance may support positive body image and, potentially, lower disordered eating risk—the rationale for measuring both body connection and body surveillance in this study.

### 1.4 Movement-based and body-oriented interventions

Eating-disorder prevention has a strong cognitive and dissonance-based tradition, exemplified by programs that reduce internalization of the thin ideal and yield reductions in risk (Stice et al., 2006). Meta-analytic evidence indicates that such prevention programs can reduce eating-disorder risk factors and, in some trials, the onset of eating disorders (Stice et al., 2021). More recent controlled trials have extended this tradition by tailoring dissonance-based and functionality-focused programs to adult and non-Western women, including the Expand Your Horizon and Women’s Body Initiative protocols and a school-based intervention in India (Toole et al., 2021; Verzijl et al., 2021; Davies et al., 2022; Lewis-Smith et al., 2023), and a systematic review and meta-analysis confirms that randomized psychosocial interventions can improve body image among non-clinical women (Zhao et al., 2025). Body-oriented and movement-based approaches are increasingly viewed as valuable complements to these cognitive programs, because they engage the body directly rather than only its cognitive representation (Alleva and Tylka, 2021).

Among these, somatic orientations within dance movement therapy (DMT) and related body-awareness movement programs have accumulated meta-analytic support for benefits to body image and related psychological outcomes (Koch et al., 2019). Recent quasi-experimental and controlled studies in young women, including team-sport and functional-training programs, have extended this evidence to non-clinical campus populations using the same family of measures employed here, namely body appreciation, embodiment, functionality appreciation, and body-surveillance indices (Jankauskiene et al., 2024; Zhu and Wen, 2025).

Despite this progress, the somatic dance course studied here—a non-competitive, somatic-oriented course centered on improvisational kinesthetic exploration—has rarely been tested as a structured campus program that simultaneously evaluates body appreciation, embodied self-awareness, and disordered eating risk. Its proposed mechanism is therefore not simply that students move more. Unlike codified performance-oriented movement training, the course does not ask participants to display an aesthetically correct body or movement form; unlike competitive sport, it does not organize bodily experience around ranking, winning, or technical evaluation; and unlike standard fitness programs, it places body-function awareness inside a reflective practice of interoceptive attention, bodily boundaries, agency, and non-judgmental group discussion. The course also does not depend on fixed postures or prescribed breathing routines; its distinctive component is improvisational kinesthetic exploration. This represents a clear gap, given that the somatic dance course maps closely onto the protective mechanisms identified by embodiment and objectification theories: it is intended to shift attention away from appearance surveillance and toward internally sensed body connection, functional appreciation, and embodied self-care.

### 1.5 The present study

Addressing this gap, the present study evaluated an 8-week somatic dance course for female university students within a quasi-experimental, pre-post controlled design. Grounded in the developmental theory of embodiment (Piran, 2016) and objectification theory (Fredrickson and Roberts, 1997), and using validated indicators of positive body image and its correlates (Tylka and Wood-Barcalow, 2015a; Alleva et al., 2017; Price and Thompson, 2007), we examined five outcomes: body appreciation, embodied self-awareness, disordered eating risk, functionality appreciation, and body surveillance.

We hypothesized that, relative to a comparison group attending regular university physical education electives, the somatic dance group would show greater increases in the primary outcome of body appreciation and in the secondary outcomes of embodied self-awareness and functionality appreciation, and greater decreases in the secondary outcome of body surveillance (H1). We also examined whether disordered eating risk, a secondary outcome expected to be relatively low at baseline in this non-clinical sample, would decline more in the intervention group (H2). Exploratory analyses examined whether change in embodied self-awareness was indirectly associated with changes in the other outcomes (H3). The proposed conceptual model linking the somatic dance course, embodied self-awareness, and body-image outcomes is presented in Figure 1.

**Figure 1:**
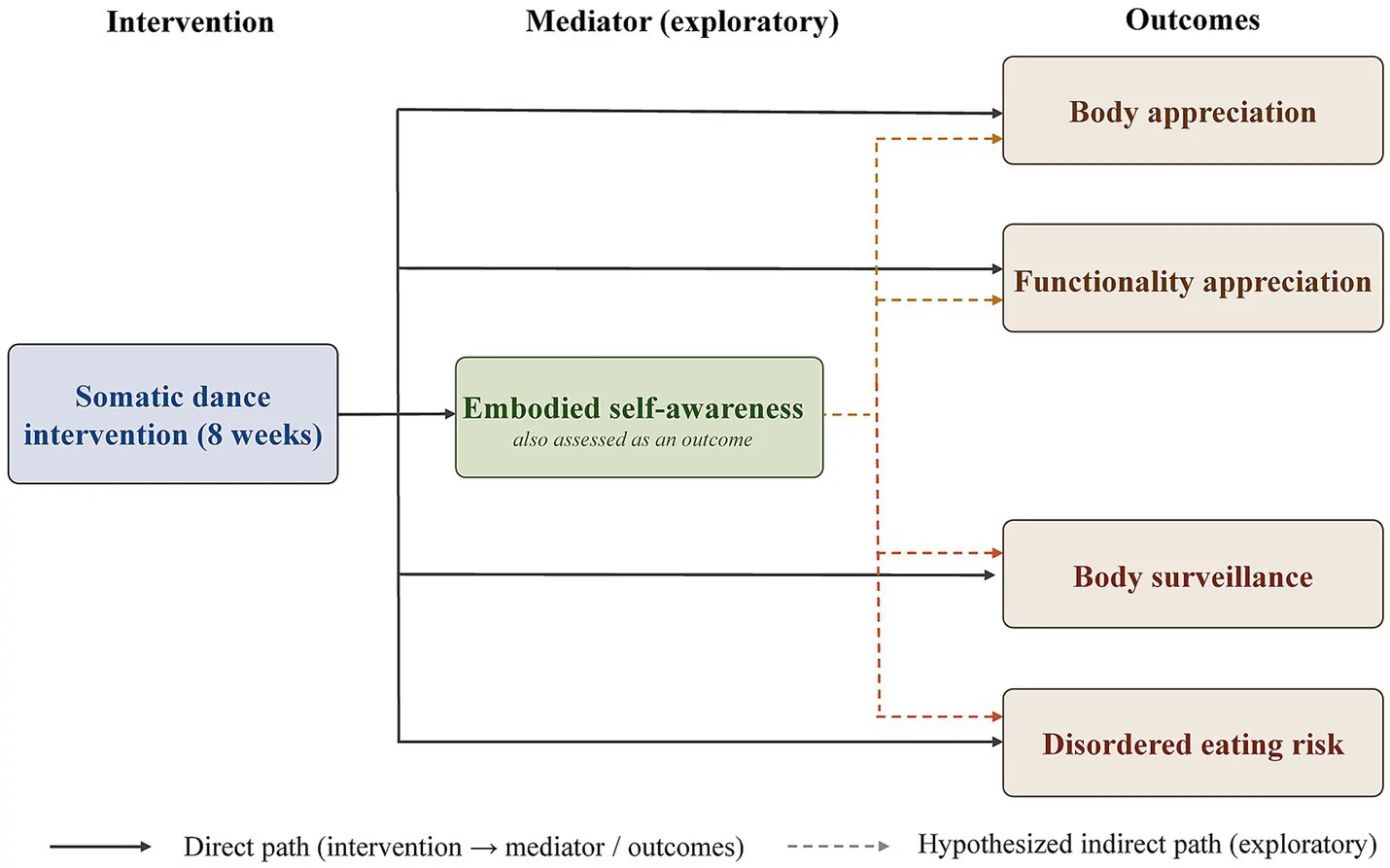
Theoretical model of the somatic dance course, showing the intervention, embodied self-awareness as the exploratory mediator, and the five measured outcomes. Exploratory mediation analyses tested only change in embodied self-awareness as the mediator; functionality appreciation and body surveillance are presented as outcomes rather than as tested mediators.

## 2 Materials and methods

### 2.1 Study design

This study employed a quasi-experimental, pre-post controlled design to examine changes associated with an 8-week somatic-oriented improvisational dance course in body image, embodied self-awareness, and disordered eating risk in female university students. Because allocation followed students’ existing enrollment in university elective physical education courses rather than individual randomization, the design is best characterized as a non-randomized controlled intervention study, consistent with prior body-image intervention research in young women (Jankauskiene et al., 2024). Participants were assigned either to an experimental group, which received the non-competitive somatic dance course, or to a comparison group, which attended regular university physical education electives.

The study was conducted within a university elective physical education curriculum during the autumn semester of the 2025/2026 academic year. The intervention course was framed as non-competitive body education and centered on somatic-oriented improvisational movement rather than performance-oriented training. Baseline (pre-test) assessments were administered in the week preceding the first session, the intervention was delivered across the subsequent 8 weeks (Weeks 1–8), and post-intervention (post-test) assessments were completed in Week 9. No follow-up assessment was conducted; accordingly, the present study examined short-term changes rather than the long-term prevention of disordered eating. The study was conducted in accordance with the Declaration of Helsinki. At the time of conduct, the institutional research ethics committee determined that the study was exempt from mandatory ethics review, as it constituted minimal-risk, non-clinical, anonymous questionnaire-based research with voluntary participation and written informed consent. Approval No. XZIT-IRB-2026-001, dated 7 July 2026, was subsequently issued to document that determination. All participants provided written informed consent before enrollment. The flow of participants through the study is summarized in Figure 2.

**Figure 2:**
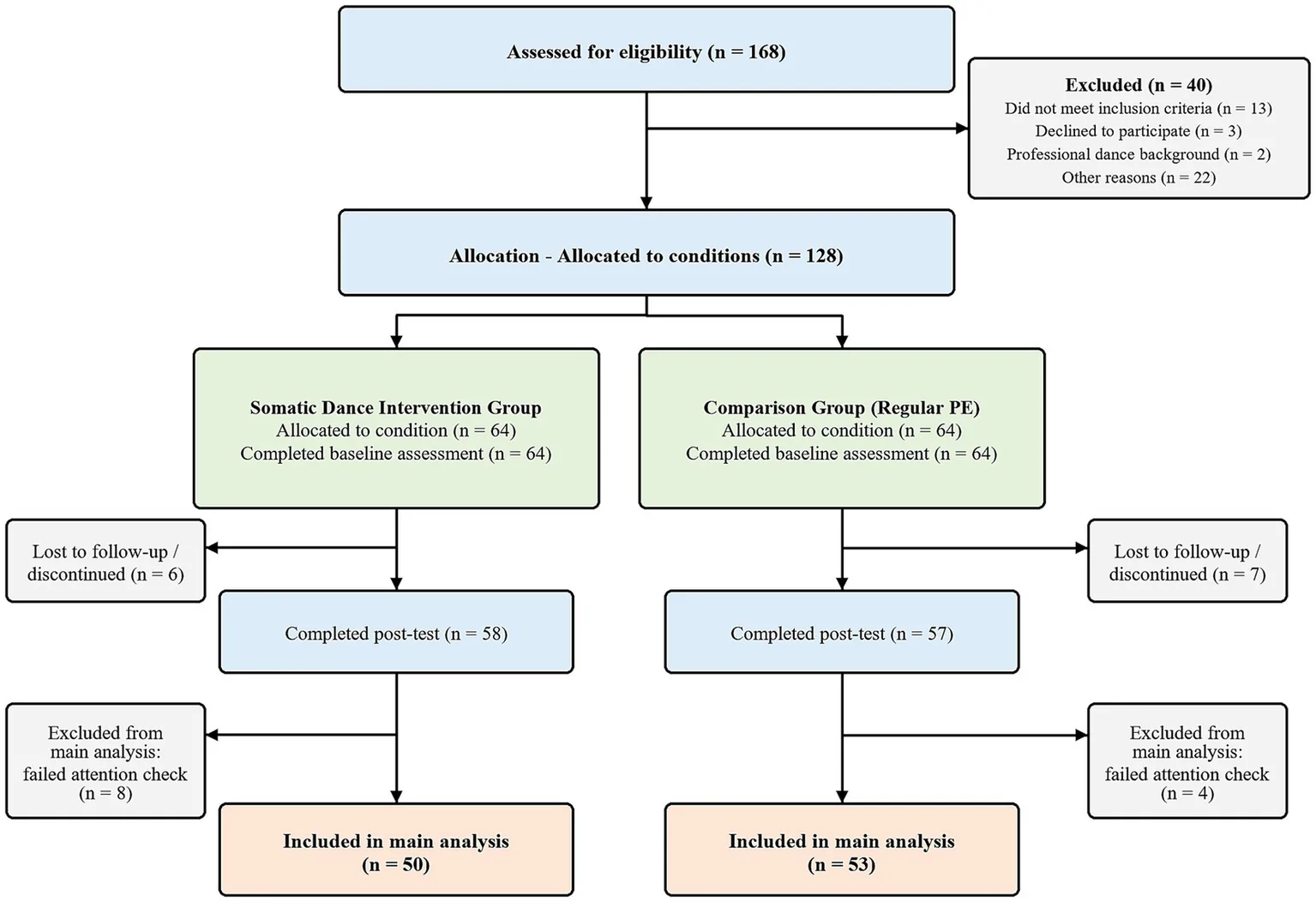
Participant flow diagram showing enrollment, eligibility screening, allocation, completion of pre- and post-intervention assessments, attention-check exclusions, and inclusion in the main analysis sample.

### 2.2 Participants and recruitment

Participants were recruited from female undergraduate students enrolled in university elective physical education courses at a comprehensive university in China during September 2025. Recruitment was conducted through course-registration announcements, campus notices, and brief in-class presentations. Students who expressed interest completed an online screening questionnaire used to determine eligibility. A two-group planning calculation conducted in G*Power 3.1, assuming equal allocation, a medium effect (Cohen’s d = 0.50), 80% statistical power, and a two-sided significance level of 0.05, indicated that approximately 64 participants per group (128 total) would be required before attrition or exclusions. This effect size was used as a planning assumption and was not estimated from the present data. We therefore sought to enroll the full available course cohort, with a target of approximately 128 allocated participants; the final effective sample was interpreted with attention to precision because post-test non-completion, attention-check exclusions, and per-outcome missingness reduced the analytic n below this planning target.

Inclusion criteria were: (a) female undergraduate students aged 18–25 years; (b) willingness and availability to complete the full 8-week course; (c) provision of written informed consent; and (d) the absence of medical contraindications to moderate physical activity. Screening excluded students with: (a) a current diagnosis of a clinical eating disorder under active specialized treatment, as the course was a non-clinical preventive elective rather than a substitute for specialized treatment; (b) clearly identified professional or advanced training in codified movement forms, or regular structured mind–body movement practice during the preceding 3 months; (c) current enrollment in a structured weight-loss program; (d) acute injury or other conditions precluding movement-based activity; and (e) an acute psychological crisis requiring immediate clinical care. Baseline questionnaires also used broader background items to record prior structured dance or movement experience and recent structured mind–body movement exposure among enrolled participants. These residual baseline reports were self-reported after screening, did not necessarily meet the screening exclusion threshold, and were retained in the main analysis and examined in sensitivity analyses. Detailed eligibility, allocation, and retention are reported in Section 3.1 and Figure 2.

### 2.3 Intervention and comparison conditions

#### 2.3.1 Somatic dance course

The somatic dance course was a manualized 8-week program designed to foster positive body image and embodied self-awareness by redirecting attention from external appearance evaluation toward internal bodily sensation, functional appreciation, and bodily agency. The program was grounded in the developmental theory of embodiment (Piran, 2016) and in principles of somatic movement education, and was operationalized so that each weekly theme mapped onto a specific theoretical target and a corresponding study outcome (Table 1).

**Table 1:** Structure and theoretical targets of the 8-week somatic dance course.

Week	Theme	Primary activities and focus	Theoretical target	Targeted outcome
1	Safety and boundaries	Establishing a safe, non-judgmental space; comfortable movement and body boundaries	Embodied safety	Embodied self-awareness
2	Breath and grounding	Awareness of breath and weight; stability and bodily support	Body connection	Embodied self-awareness
3	Non-evaluative movement	Movement without appearance monitoring or technical evaluation; process over product	Reduced surveillance	Body surveillance
4	Functionality appreciation	Exploring what the body can do—strength, flexibility, coordination, balance	Functional valuing	Functionality appreciation
5	Emotion and bodily cues	Recognizing emotion through bodily sensation; movement-based expression and regulation	Interoceptive awareness	Embodied self-awareness
6	Body boundaries and agency	Choice-making in movement; resisting the external gaze; reclaiming autonomy	Bodily agency	Body surveillance
7	Relational embodiment	Partner and group movement emphasizing connection, trust, and shared experience	Supportive embodiment	Body appreciation
8	Integration and self-care	Synthesizing body-awareness practices; planning continued embodied self-care	Integration	Body appreciation

The intervention comprised 16 sessions (two 60-min sessions per week) delivered over 8 weeks. Each session followed a fixed three-part structure: interoceptive awareness practice (5 min), non-judgmental movement exploration (40 min), and group sharing and body journaling (15 min). Full session plans and instructor scripts are provided in Supplementary materials 1, 2.

The intervention comprised 16 sessions delivered over 8 weeks, with two 60-min sessions per week, for a total of 960 min of contact time. Sessions were conducted in a university movement studio without mirrors, in order to minimize external appearance monitoring, and followed a standardized instructor manual with detailed session plans and scripted verbal cues (Supplementary materials 1, 2). The program was delivered by a certified somatic movement educator who completed a structured training workshop on the protocol, its theoretical foundations, and the ethical considerations of working with body-image concerns prior to implementation.

Each session followed a consistent three-part structure: (a) interoceptive awareness practice (5 min), comprising a guided body scan directing attention to internal sensations such as breath, heartbeat, muscle tension, and temperature without evaluation; (b) non-judgmental movement exploration (40 min), comprising participant-directed improvisational movement emphasizing bodily sensation, functional capacity, and self-expression, during which any language referring to weight, body shaping, appearance, technical perfection, or performance ranking was explicitly prohibited; and (c) group sharing and body journaling (15 min), comprising facilitated reflection and structured journal entries. The eight weekly themes progressed from safety and boundaries (Week 1) through breath and grounding, non-evaluative movement, functionality appreciation, emotion and bodily cues, body boundaries and agency, and relational embodiment, to integration and self-care (Week 8); the full structure and its theoretical targets are detailed in Table 1.

#### 2.3.2 Comparison condition

Participants in the comparison group attended their regular university physical education elective courses over the same period and with the same scheduling (8 weeks, twice weekly, 60 min per session). These courses were three standard curricular electives: rouli qiu (a traditional Chinese racket-based fitness activity; *n* = 22), basketball (*n* = 22), and swimming (*n* = 20). Each ran for 16 sessions over the same 8-week period. These courses comprised conventional sport and fitness activities; courses centered on body-shaping or structured mind–body practice were not eligible for the comparison condition. Comparison-group instructors were blind to the study hypotheses and delivered standard curricular content focused on skill acquisition and fitness. Attendance was recorded for both groups. Session-level content was not systematically recorded for the comparison condition.

### 2.4 Measures

All outcome measures were administered through secure online questionnaires at baseline (Week 0) and post-intervention (Week 9). At each assessment, the questionnaire included one attention-check item requiring participants to select 3; any response other than 3 was classified as a failed check. Validated Chinese versions were used when available; for measures without a published Chinese validation source identified for the present context, items were translated and back-translated by bilingual researchers, with discrepancies resolved by discussion. Internal consistency for each scale in the present sample is reported below as Cronbach’s α at baseline and post-intervention, and additional exploratory psychometric checks are provided in Supplementary Table S1. Confirmatory factor analysis (CFA) fit indices and standardized loadings are reported in Supplementary Table S1. Fit indices varied across scales and time points; the lowest values were observed for the EAT-26 at post-intervention (CFI = 0.905; TLI = 0.892; lowest standardized loading = 0.32), so findings for disordered eating risk are interpreted cautiously.

#### 2.4.1 Body appreciation

Body appreciation was assessed with the Chinese version of the Body Appreciation Scale-2 (BAS-2; Tylka and Wood-Barcalow, 2015a; Ma et al., 2022). The BAS-2 comprises 10 items capturing acceptance of and respect for the body irrespective of appearance, rated on a 5-point scale ranging from 1 (never) to 5 (always); the total score is the item mean, with higher scores indicating greater body appreciation. In the present study, Cronbach’s α was 0.88 at baseline and 0.90 at post-intervention.

#### 2.4.2 Embodied self-awareness

Embodied self-awareness was operationalized as body connection and assessed with a Chinese adaptation of the Scale of Body Connection (SBC; Price and Thompson, 2007), which captures awareness of, and connection to, internal bodily states. Because no published Chinese validation source was identified for the present context, items were translated and back-translated as described above. Items were rated on a 5-point scale; after reverse-coding so that higher scores reflect greater body connection and lower bodily dissociation, higher mean scores indicate stronger embodied self-awareness. Cronbach’s α was 0.82 at baseline and 0.86 at post-intervention.

#### 2.4.3 Disordered eating risk

Disordered eating risk was assessed with the Chinese version of the Eating Attitudes Test-26 (EAT-26; Garner et al., 1982; Kang et al., 2017), a widely used screening instrument for disordered eating attitudes and behaviors. Following standard scoring, total scores range from 0 to 78, with higher scores indicating more pronounced disordered eating attitudes. A score of 20 or above was used as the conventional EAT-26 threshold for elevated screening risk to maintain comparability with the broader EAT-26 literature; however, lower Chinese clinical cutoffs of 14–15 have also been reported (Kang et al., 2017), so threshold-based baseline-risk estimates using 20 should be interpreted as conservative. Throughout, this construct is described as disordered eating attitudes or disordered eating risk and is not interpreted as a clinical eating-disorder diagnosis. Cronbach’s α was 0.85 at baseline and 0.83 at post-intervention.

#### 2.4.4 Functionality appreciation

Functionality appreciation was assessed with a Chinese adaptation of the Functionality Appreciation Scale (FAS; Alleva et al., 2017), comprising 7 items that capture appreciation for what the body is able to do. Because no peer-reviewed Chinese validation source was identified for the present context, items were translated and back-translated as described above. Items were rated on a 5-point scale from 1 (strongly disagree) to 5 (strongly agree), with higher mean scores reflecting greater functionality appreciation. Cronbach’s α was 0.87 at baseline and 0.89 at post-intervention.

#### 2.4.5 Body surveillance

Body surveillance, indexing self-objectification and habitual monitoring of outward appearance, was assessed with a Chinese adaptation of the Body Surveillance subscale of the Objectified Body Consciousness Scale (OBCS; McKinley and Hyde, 1996), comprising 8 items rated on a 7-point scale from 1 (strongly disagree) to 7 (strongly agree). Because no published Chinese validation source was identified for the present context, items were translated and back-translated as described above. Higher mean scores indicate more frequent appearance monitoring. Cronbach’s α was 0.79 at baseline and 0.84 at post-intervention.

#### 2.4.6 Covariates and process variables

At baseline, participants reported age, height and weight (used to compute body mass index, BMI), academic year, major, prior structured dance or movement experience, recent structured mind–body movement exposure, current dieting or weight-control behavior, and habitual physical activity. Process variables comprised session-level attendance, any adverse events, and post-intervention course satisfaction; optional open comments provided descriptive feedback on participants’ experiences. Baseline characteristics by group are summarized in Table 2.

**Table 2:** Baseline characteristics of participants by group.

Characteristic	Experimental (*n* = 50)	Comparison (*n* = 53)	*p*	SMD
Age (years), M ± SD	19.91 ± 1.01	20.32 ± 1.29	0.077	−0.35
BMI (kg/m^2^), M ± SD	21.32 ± 2.23	20.62 ± 2.36	0.126	0.31
BAS-2 (1–5), M ± SD	3.07 ± 0.53	3.17 ± 0.50	0.332	−0.19
SBC, reverse-scored (1–5), M ± SD	3.19 ± 0.42	3.23 ± 0.45	0.701	−0.08
EAT-26 total (0–78), M ± SD	10.62 ± 5.16	8.71 ± 5.66	0.084	0.35
FAS (1–5), M ± SD	3.28 ± 0.50	3.12 ± 0.47	0.108	0.33
OBCS-BS (1–7), M ± SD	4.26 ± 0.81	4.27 ± 0.66	0.922	−0.02
Academic year, *n* (%)			0.892	0.13
First year	14 (28.0)	14 (26.4)		
Second year	10 (20.0)	13 (24.5)		
Third year	14 (28.0)	16 (30.2)		
Fourth year	12 (24.0)	10 (18.9)		
Baseline-reported dance/movement experience, *n* (%)			0.698	0.20
None	33 (66.0)	31 (58.5)		
Occasional	10 (20.0)	13 (24.5)		
School club / elective	7 (14.0)	8 (15.1)		
Formal/advanced training (self-reported)	0 (0.0)	1 (1.9)		
Baseline-reported structured mind–body movement exposure, *n* (%)			0.740	0.15
None	29 (58.0)	33 (62.3)		
Occasional	15 (30.0)	16 (30.2)		
Regular exposure, self-reported (past 3 months)	6 (12.0)	4 (7.5)		
Baseline EAT-26 ≥ 20, *n* (%)	5 (10.0)	3 (5.7)	0.480	0.16

BMI, body mass index; BAS-2, Body Appreciation Scale-2; SBC, Scale of Body Connection (embodied self-awareness); EAT-26, Eating Attitudes Test-26; FAS, Functionality Appreciation Scale; OBCS-BS, Objectified Body Consciousness Scale–Body Surveillance subscale; M, mean; SD, standard deviation; SMD, standardized mean difference (experimental minus comparison). Continuous variables were compared using Welch’s t-test and categorical variables using Fisher’s exact or chi-square tests.

### 2.5 Intervention fidelity

Several procedures supported consistent delivery. The instructor followed a session-by-session manual with standardized activity descriptions, timing guidance, and verbatim scripts for key prompts (Supplementary materials 1, 2). After each session, the instructor completed a fidelity checklist documenting adherence to four core components: completion of the interoceptive awareness practice, maintenance of non-judgmental language (no comments on appearance, weight, or shape), completion of body journaling and group sharing, and adherence to the session script (Supplementary material 3). Protocol deviations, adverse events, and an observer-rated 0–100 global fidelity score were recorded. The observations were conducted by two recorders who were not involved in delivering the course, one serving as the primary recorder and the other as backup, and session records were usually agreed through discussion between them. No formal inter-rater reliability statistic was calculated. Indices of fidelity across the 16 sessions are summarized in Supplementary material 3 and reported quantitatively in Section 3.2.

### 2.6 Statistical analysis

Analyses were conducted in Python (version 3.9) using the statsmodels, scipy, and pingouin libraries. Confirmatory factor analyses were conducted in R (version 4.3.3) using lavaan; methodological details are provided in Supplementary Table S1. Statistical significance was set at *p* < 0.05 (two-tailed). Effect sizes are reported as Hedges’ g, which corrects for small-sample bias, with values of approximately 0.20, 0.50, and 0.80 interpreted as small, medium, and large, respectively.

#### 2.6.1 Preliminary analyses

Data-cleaning procedures addressed duplicate submissions, item-level missingness, and attention-check failures. Participants who completed at least 80% of the items on a scale were retained. For BAS-2, SBC, FAS, and OBCS-BS, scale scores were calculated as the mean of observed items; for EAT-26, one missing item was replaced by the median of the available scored items, rounded up to a whole-number item score, before calculating the conventional 0–78 total score, whereas more than one missing item resulted in a missing EAT-26 score. Participants who failed an attention check at either time point were excluded from the primary analysis but retained for sensitivity analysis. Descriptive statistics were computed for all variables, and baseline group differences were examined using Welch’s independent-samples t-tests for continuous variables and Fisher’s exact or chi-square tests for categorical variables. Standardized mean differences (SMDs) were calculated to evaluate baseline balance, and internal consistency was assessed with Cronbach’s α. For the Chinese versions and adaptations used in the present study, exploratory psychometric checks also summarized McDonald’s omega, corrected item-total correlations, and basic CFA fit indices; these checks document the measurement performance of the scales in this sample.

#### 2.6.2 Primary and secondary group comparisons

Body appreciation (BAS-2) was designated as the primary outcome, consistent with the sample-size planning. Secondary outcomes were embodied self-awareness (SBC), disordered eating risk (EAT-26), functionality appreciation (FAS), and body surveillance (OBCS-BS). Given the quasi-experimental design and the possibility of residual baseline differences, group differences were estimated using analysis of covariance (ANCOVA), which is preferred over the simple comparison of change scores in controlled pre-post designs (Vickers and Altman, 2001). For each outcome, the post-intervention score served as the dependent variable, group (experimental versus comparison) as the predictor, and the corresponding baseline score together with baseline BMI as covariates. Models yielded adjusted group means, adjusted mean differences with 95% confidence intervals (CIs), and *p*-values; Hedges’ g was derived from adjusted means and pooled standard deviations. The main analysis dataset comprised participants who passed attention checks at both time points (*n* = 103). Because descriptive summaries and ANCOVA models used outcome-specific complete cases, and because the ANCOVA models additionally required baseline BMI, the effective n varied by outcome; main ANCOVA complete-case n ranged from 93 to 100. One participant in the main analysis sample lacked baseline BMI and was excluded from the ANCOVA models. For disordered eating risk, a check in which BMI was removed as a covariate gave a directionally consistent result. Model assumptions were examined using residual diagnostics and Levene’s test. Because only body appreciation was designated as the primary outcome, the secondary outcomes, sensitivity analyses, and exploratory mediation paths were interpreted with emphasis on effect sizes, confidence intervals, and consistency of the overall pattern rather than as independently confirmatory tests; *p*-values were not adjusted for multiplicity.

#### 2.6.3 Sensitivity analyses

Sensitivity analyses evaluated the robustness of the main findings using: (a) all matched participants regardless of attention-check performance; (b) a per-protocol analysis restricting participants from both groups to those who passed both attention checks and attended at least 75% of the 16 sessions (at least 12 sessions), an operational threshold adopted for this sensitivity analysis; and (c) exclusion of participants with residual baseline reports of prior formal dance or movement training or recent regular structured mind–body movement exposure. Each sensitivity model replicated the main ANCOVA specification. Because academic major differed between groups at baseline, an additional *post hoc* robustness model repeated the main ANCOVA with academic major category entered as a categorical covariate.

#### 2.6.4 Exploratory mechanism analyses

Exploratory change-score mediation analyses tested whether the group differences in body appreciation, disordered eating risk, functionality appreciation, and body surveillance were indirectly associated with change in embodied self-awareness. Models specified group as the independent variable, change in the proposed mediator (post minus pre) as the mediator, and change in the outcome as the dependent variable, with baseline mediator and outcome values and baseline BMI as covariates. Indirect effects were evaluated using 95% bias-corrected bootstrap CIs based on 10,000 resamples. Because embodied self-awareness was also measured as a study outcome and was assessed at the same pre- and post-intervention time points as the other outcomes, these analyses cannot establish temporal precedence or causal mediation. They are therefore framed as exploratory statistical mediation analyses.

## 3 Results

### 3.1 Participant flow and baseline characteristics

Of 168 students assessed for eligibility, 40 were excluded during screening, and 128 were enrolled and allocated to the experimental (*n* = 64) and comparison (*n* = 64) groups. All allocated participants received their assigned condition. Post-intervention questionnaires were completed by 58 experimental and 57 comparison participants, yielding 115 matched pre-post records (89.8% retention). After excluding 12 participants who failed an attention check at one or both time points (experimental, *n* = 8; comparison, n = 4), the main analysis sample comprised 103 participants (experimental, *n* = 50; comparison, *n* = 53). The analysis sample of 103 participants is the pool from which each analysis draws; because each outcome uses participants with complete data for that outcome, and the ANCOVA models additionally require baseline BMI, the n reported for individual outcomes is lower and varies across outcomes (Tables 3, 4). Participant flow is depicted in Figure 2.

**Table 3:** Pre- and post-intervention scores for primary and secondary outcomes.

Outcome	Group	*n*	Pre, M ± SD	Post, M ± SD	Change, M ± SD
Body appreciation (BAS-2)	Experimental	48	3.06 ± 0.53	3.50 ± 0.59	0.44 ± 0.46
	Comparison	52	3.17 ± 0.50	3.29 ± 0.58	0.13 ± 0.47
Embodied self-awareness (SBC)	Experimental	49	3.19 ± 0.42	3.55 ± 0.50	0.36 ± 0.37
	Comparison	52	3.23 ± 0.45	3.37 ± 0.44	0.14 ± 0.38
Disordered eating risk (EAT-26)	Experimental	44	10.73 ± 5.32	8.93 ± 4.87	−1.80 ± 5.44
	Comparison	50	8.68 ± 5.72	8.92 ± 4.14	0.24 ± 5.90
Functionality appreciation (FAS)	Experimental	47	3.29 ± 0.50	3.76 ± 0.52	0.48 ± 0.39
	Comparison	51	3.11 ± 0.48	3.28 ± 0.61	0.17 ± 0.49
Body surveillance (OBCS-BS)	Experimental	49	4.25 ± 0.82	3.92 ± 1.07	−0.34 ± 0.65
	Comparison	51	4.25 ± 0.65	4.26 ± 0.76	0.00 ± 0.67

BAS-2, FAS, and SBC are scored 1–5 with higher scores indicating more favorable body image; SBC (Scale of Body Connection) is reverse-scored so that higher values reflect stronger embodied self-awareness. EAT-26 is scored 0–78 and OBCS-BS 1–7, with lower scores indicating lower disordered eating risk and lower body surveillance, respectively. Change scores are post minus pre. Within each outcome-by-group row, n, pre-test, post-test, and change values were calculated from the same attention-check-passing participants with complete pre- and post-test scale data for that outcome. Adjusted analyses (Table 4) use complete cases per outcome with the corresponding baseline outcome and baseline BMI covariates. One attention-check-passing participant lacked baseline BMI, so Table 4 n values are one lower than the corresponding table descriptive *n* values where that participant otherwise had complete scale data.

**Table 4:** Adjusted group differences and sensitivity analyses.

Sample	Outcome	*n*	Adj. diff.	95% CI	*p*	*g*
Main analysis	Body appreciation (BAS-2)	99	0.31	0.13, 0.49	<0.001	0.69
(Attention-pass)	Embodied self-awareness (SBC)	100	0.23	0.10, 0.37	0.001	0.67
	Disordered eating risk (EAT-26)	93	−0.54	−2.29, 1.21	0.542	−0.13
	Functionality appreciation (FAS)	97	0.35	0.18, 0.53	<0.001	0.82
	Body surveillance (OBCS-BS)	99	−0.36	−0.63, −0.09	0.009	−0.54
Major-adjusted	Body appreciation (BAS-2)	99	0.31	0.10, 0.52	0.004	0.66
	Embodied self-awareness (SBC)	100	0.21	0.06, 0.36	0.007	0.61
	Disordered eating risk (EAT-26)	93	−0.57	−2.56, 1.42	0.572	−0.13
	Functionality appreciation (FAS)	97	0.33	0.12, 0.53	0.002	0.74
	Body surveillance (OBCS-BS)	99	−0.41	−0.72, −0.10	0.010	−0.60
All matched	Body appreciation (BAS-2)	109	0.37	0.19, 0.54	<0.001	0.80
	Embodied self-awareness (SBC)	110	0.23	0.09, 0.36	0.001	0.63
	Disordered eating risk (EAT-26)	102	−1.39	−3.09, 0.31	0.109	−0.32
	Functionality appreciation (FAS)	107	0.34	0.17, 0.51	<0.001	0.77
	Body surveillance (OBCS-BS)	107	−0.39	−0.64, −0.14	0.003	−0.60
Per-protocol	Body appreciation (BAS-2)	76	0.32	0.10, 0.54	0.004	0.69
(≥75% attend.)	Embodied self-awareness (SBC)	77	0.29	0.14, 0.45	<0.001	0.86
	Disordered eating risk (EAT-26)	72	0.30	−1.64, 2.23	0.760	0.07
	Functionality appreciation (FAS)	75	0.25	0.06, 0.43	0.009	0.63
	Body surveillance (OBCS-BS)	78	−0.34	−0.64, −0.03	0.033	−0.50
Excl. baseline-reported	Body appreciation (BAS-2)	88	0.32	0.13, 0.51	0.001	0.72
Dance/mind–body movement exposure	Embodied self-awareness (SBC)	89	0.22	0.07, 0.37	0.004	0.63
	Disordered eating risk (EAT-26)	82	−0.27	−2.14, 1.61	0.777	−0.06
	Functionality appreciation (FAS)	86	0.38	0.18, 0.58	<0.001	0.83
	Body surveillance (OBCS-BS)	88	−0.33	−0.62, −0.04	0.026	−0.49

Adj. diff., adjusted mean difference (experimental minus comparison) from ANCOVA models with the corresponding baseline outcome and baseline BMI as covariates; CI, confidence interval; g, Hedges’ g. The major-adjusted robustness model additionally includes academic major category as a categorical covariate. The baseline-exposure exclusion model removed participants with residual baseline reports of prior formal dance or movement training or recent regular structured mind–body movement exposure. Models used outcome-specific complete cases with non-missing baseline BMI; the main attention-check-passing dataset included up to 103 participants, and main ANCOVA effective n ranged from 93 to 100. Abbreviations as in Table 3.

Baseline characteristics of the main analysis sample are presented in Table 2. The groups did not differ significantly in age (experimental: *M* = 19.91, SD = 1.01; comparison: *M* = 20.32, SD = 1.29; *p* = 0.077) or BMI (experimental: *M* = 21.32, SD = 2.23; comparison: *M* = 20.62, SD = 2.36; *p* = 0.126), and academic-year distribution was comparable (*p* = 0.892). Standardized mean differences for the baseline outcome measures ranged from −0.19 to 0.35 (Table 2); the largest were for the EAT-26 (0.35), reflecting higher baseline disordered eating attitudes in the experimental group, and the FAS (0.33). The groups differed in the distribution of academic majors (*p* = 0.027), with relatively more experimental-group students majoring in physical education and psychology and relatively more comparison-group students in nursing and computer science. Prior structured dance or movement experience and recent structured mind–body movement exposure did not differ between groups (both *p* > 0.69). At baseline, 8 participants (7.8% of the main analysis sample; 8.2% of those with valid baseline EAT-26 scores) scored at or above the conventional EAT-26 threshold of 20 (experimental, *n* = 5, 10.0%; comparison, *n* = 3, 5.7%; *p* = 0.480). Using lower Chinese clinical cutoffs reported by Kang et al. (2017) would yield higher elevated-risk proportions (≥15: 18/98, 18.4%; ≥14: 21/98, 21.4%), so the threshold-based baseline-risk estimate should be viewed as conservative.

### 3.2 Intervention adherence and fidelity

Experimental-group post-test completers attended on average 13.45 of 16 sessions (SD = 1.79; range 8–16), corresponding to 84.1% attendance; 52 of the 58 post-test completers (89.7%) attended at least 75% of sessions. Among experimental-group participants retained in the main attention-check sample, 44 of 50 (88.0%; 68.8% of those allocated) met the per-protocol criterion. Comparison-group post-test completers attended on average 12.49 sessions (SD = 1.95; range 8–16) of their regular university physical education electives. Intervention fidelity was high overall. Interoceptive awareness practices and non-judgmental movement principles were implemented across sessions, while protocol deviations, mostly minor timing or setting adjustments, were documented in 7 of 16 sessions; appearance- or weight-related language was also documented and redirected according to the protocol. The mean fidelity score rated by the two recorders was 91.3 out of 100 (session-level range 62–100), with the lowest score occurring in an early session involving shortened sharing/journaling, script deviation, and redirected appearance-related language (Supplementary material 3). No adverse events requiring medical referral or study discontinuation occurred in either group. Among experimental-group post-test completers, 57 of 58 provided course-satisfaction ratings; the mean rating was 4.02 (SD = 0.61) on a 1–5 scale, and 47 of 57 participants (82.5%) rated the course 4 or 5.

### 3.3 Primary and secondary outcomes

Descriptive statistics for all outcomes at pre- and post-intervention are presented in Table 3, and adjusted group differences from the ANCOVA models are presented in Table 4. For the primary outcome, body appreciation showed a larger adjusted post-intervention difference favoring the experimental group: after adjustment for baseline BAS-2 and BMI, the adjusted group means were 3.56 and 3.25, respectively, with an adjusted difference of 0.31 (95% CI: 0.13, 0.49; *p* < 0.001; Hedges’ *g* = 0.69), a medium-to-large effect. Among the secondary outcomes, embodied self-awareness also favored the experimental group (adjusted means 3.59 versus 3.35; adjusted difference 0.23, 95% CI: 0.10, 0.37; *p* = 0.001; *g* = 0.67), a medium effect. In contrast, although disordered eating risk decreased numerically more in the experimental group (change *M* = −1.80) than in the comparison group (change *M* = 0.24), the adjusted between-group difference was not statistically significant (adjusted difference −0.54, 95% CI: −2.29, 1.21; *p* = 0.542; *g* = −0.13). Sample-size planning targeted body appreciation as the primary outcome, and the present analysis of disordered eating risk had limited power to detect small between-group differences; the confidence interval for this outcome includes both no difference and a reduction of approximately two points on the EAT-26 total score.

The remaining secondary outcomes followed the same general pattern. Functionality appreciation favored the experimental group (adjusted means 3.69 versus 3.34; adjusted difference 0.35, 95% CI: 0.18, 0.53; *p* < 0.001; *g* = 0.82), a large effect, and body surveillance was lower in the experimental group (adjusted means 3.88 versus 4.24; adjusted difference −0.36, 95% CI: −0.63, −0.09; *p* = 0.009; *g* = −0.54), a medium effect. Residual diagnostics, Levene’s tests, and checks of group-by-covariate interactions did not indicate material violations of ANCOVA assumptions for the main models.

### 3.4 Sensitivity analyses

Sensitivity analyses, summarized in Table 4, supported the robustness of the main findings. When all matched participants were included regardless of attention-check performance, the group differences remained significant and showed a similar overall pattern for body appreciation (adjusted difference 0.37; *p* < 0.001; *g* = 0.80), embodied self-awareness (0.23; *p* = 0.001; *g* = 0.63), functionality appreciation (0.34; *p* < 0.001; *g* = 0.77), and body surveillance (−0.39; *p* = 0.003; *g* = −0.60), while the group difference in disordered eating risk remained non-significant (*p* = 0.109). In the per-protocol analysis restricting the analysis to participants in both groups who passed both attention checks and attended at least 75% of sessions, group differences again reached significance for body appreciation (0.32; *p* = 0.004; *g* = 0.69), embodied self-awareness (0.29; *p* < 0.001; g = 0.86), functionality appreciation (0.25; *p* = 0.009; *g* = 0.63), and body surveillance (−0.34; *p* = 0.033; *g* = −0.50), but not for disordered eating risk (*p* = 0.760). Excluding participants with residual baseline reports of prior formal dance or movement training or recent regular structured mind–body movement exposure produced the same pattern. In a *post hoc* robustness model additionally adjusting for academic major category, the pattern was again unchanged: body appreciation (adjusted difference 0.31; *p* = 0.004), embodied self-awareness (0.21; *p* = 0.007), functionality appreciation (0.33; *p* = 0.002), and body surveillance (−0.41; *p* = 0.010) remained significant, whereas disordered eating risk remained non-significant (*p* = 0.572).

### 3.5 Exploratory mechanism analyses

Exploratory change-score mediation analyses examined whether change in embodied self-awareness statistically accounted for the group differences in the other outcomes (*n* = 93–98 across models). Across models, the intervention group showed significantly greater change in embodied self-awareness (path a in the range 0.21–0.24; all *p* ≤ 0.004). However, change in embodied self-awareness was not significantly associated with change in any outcome after adjustment for group and baseline values (all path-b *p* ≥ 0.09). Consequently, the indirect effects were small and their 95% bootstrap CIs all included zero, for body appreciation (indirect effect 0.002; 95% CI: −0.063, 0.066), disordered eating risk (0.087; 95% CI: −0.479, 0.794), functionality appreciation (−0.043; 95% CI: −0.112, 0.009), and body surveillance (−0.000; 95% CI: −0.068, 0.079). These analyses therefore did not support an indirect pathway through change in embodied self-awareness in this sample; the data are consistent either with mechanisms operating outside the measured change-score pathway or with limited statistical power to detect indirect effects over this short interval.

## 4 Discussion

### 4.1 Principal findings

This quasi-experimental study evaluated an 8-week somatic dance course for female university students and found short-term group differences favoring the intervention in positive body image and embodiment-related outcomes; detailed adjusted estimates are reported in Section 3.3 and Table 4. The absence of a statistically significant group difference in EAT-26 scores suggests that the observed group differences were more evident for positive body-image resources than for disordered eating risk in this non-clinical sample. This result should be read with attention to statistical power: sample-size planning targeted body appreciation, and the confidence interval for disordered eating risk includes both no difference and a modest reduction. This distinction is consequential because body-image facets prospectively differentiate dietary restraint and exercise patterns and predict disordered eating in women (Raspovic et al., 2023; Anderson and Linardon, 2024). Thus, H1 was supported for the body-image and embodiment-related outcomes, whereas H2, concerning disordered eating risk, was not. The exploratory mediation hypothesis (H3) was not supported: change in embodied self-awareness did not statistically account for the group differences in the other outcomes (Section 3.5).

### 4.2 Comparison with previous intervention studies

The magnitude of the body-image differences observed here is consistent with, and in some respects exceeds, that reported for related movement-based programs. Meta-analyses of DMT and related movement interventions have documented reliable benefits for body image and related psychological outcomes (Koch et al., 2019), and randomized psychosocial interventions improve body image among non-clinical women (Zhao et al., 2025). The present group differences in body appreciation (*g* = 0.69) and functionality appreciation (g = 0.82) are comparable to those reported for functional-training programs in young women, which used the same family of measures within a non-randomized design (Jankauskiene et al., 2024), and to those of a campus basketball intervention employing embodiment and body-appreciation measures in female college students (Zhu and Wen, 2025). The gain in functionality appreciation accords with functionality-focused interventions such as Expand Your Horizon (Davies et al., 2022; Alleva and Tylka, 2021), and the lower body surveillance in the intervention group aligns with evidence that body-oriented and dissonance-based programs can shift attention from appearance monitoring toward internal bodily experience (Toole et al., 2021; Verzijl et al., 2021).

At the same time, the absence of a statistically significant group difference in disordered eating risk distinguishes the present program from the cognitive and dissonance-based prevention tradition, in which structured programs have reduced eating pathology and its risk factors (Stice et al., 2006, 2021). This divergence is interpretable: the somatic dance course was designed to build positive body-image resources rather than to directly target dietary restraint or thin-ideal internalization, and disordered eating attitudes in this non-clinical sample were low at baseline, leaving limited room for short-term change. Comparable body-image benefits without short-term change in disordered eating have been noted in other non-clinical movement and school-based programs (Lewis-Smith et al., 2023).

### 4.3 Possible mechanisms

The somatic dance group showed greater increases in embodied self-awareness, consistent with the developmental theory of embodiment. This theory holds that pleasurable, attentive movement can strengthen body connection, agency, and attuned self-care (Piran, 2016; Price and Thompson, 2007). The group also showed lower body surveillance, in line with objectification theory, which identifies habitual appearance monitoring as a process sustained by an internalized observer’s perspective (Fredrickson and Roberts, 1997; Noll and Fredrickson, 1998). Meta-analytic work confirms the centrality of body shame and self-objectification to these processes and links them to gendered and social-media-related risk contexts (Saunders et al., 2024; Guo et al., 2025; Wang et al., 2025). This pattern is compatible with evidence that internal body orientation and body connection support body appreciation and adaptive eating (Modica, 2021; Yamamiya et al., 2021), and that body appreciation predicts reductions in eating pathology (Messer et al., 2022; Linardon, 2021). Functionality appreciation is likewise associated with lower self-objectification (Golmohamadi et al., 2025; Liu et al., 2025; Ramesh et al., 2026), and self-objectification and body comparison have been implicated in disordered eating among Chinese women (Xu and Chen, 2025).

Nevertheless, the exploratory change-score mediation analyses did not support an indirect pathway through change in embodied self-awareness. Although the intervention group showed greater change in embodied self-awareness, change in this variable was not statistically associated with change in the other outcomes once group and baseline values were controlled. More fundamentally, embodied self-awareness and the other outcomes were measured at the same post-intervention time point, and embodied self-awareness was itself one of the study outcomes. The analyses therefore document whether change scores covaried in a mediation-like pattern, but they cannot establish that embodied change preceded or caused change in the other outcomes. The findings should be read as hypothesis-generating rather than as confirmation of mechanism.

### 4.4 Theoretical implications

The results add to a growing body of evidence that positive body image is a multifaceted, modifiable construct rather than the mere absence of dissatisfaction (Tylka and Wood-Barcalow, 2015b; Linardon et al., 2022). The concurrent pattern of greater body appreciation, functionality appreciation, and embodied self-awareness alongside lower body surveillance, is congruent with both the developmental theory of embodiment (Piran, 2016) and objectification theory (Fredrickson and Roberts, 1997; Saunders et al., 2024), and with evidence that functionality appreciation confers unique benefits for body image and lower self-objectification (Golmohamadi et al., 2025; Alleva and Tylka, 2021). This pattern suggests that non-aesthetic, attention-reorienting movement can engage several protective processes at once. That these gains occurred without a corresponding short-term change in disordered eating risk underscores the conceptual distinction between strengthening positive body image and reducing eating pathology, and indicates that the two may follow different timecourses.

### 4.5 Practical implications

From an applied standpoint, the program was feasible to deliver within an existing university elective body education context, achieved high attendance and fidelity, and produced no adverse events, suggesting that an embodiment-informed somatic dance course is a practical addition to campus body-image promotion. The differences reported here are at the scale-score level, and the study did not measure daily functioning, clinical outcomes, or longer-term behavior; therefore, the practical significance of these magnitudes remains to be established. Universities are well placed to offer such low-intensity, appearance-neutral movement options alongside established prevention programs (Stice et al., 2021; Koch et al., 2019). This is especially relevant given mounting evidence that social-media use, eating-related online content, and appearance pressures contribute to body-image and eating difficulties in young adults (Wu et al., 2024; Zhang et al., 2025; Yu et al., 2026), and that school- and community-based body-image interventions can be effective in non-Western and non-clinical settings (Lewis-Smith et al., 2023; Zhao et al., 2025). Because the experimental group showed a consistent pattern across multiple facets of positive body image, the somatic dance course may broaden the menu of enjoyable, body-affirming activities available to young women and complement cognitive prevention approaches rather than replace them.

### 4.6 Limitations

Several limitations define the boundary conditions of the present findings. Allocation followed students’ existing course enrollment, so assignment was not independent of their elective choices. Differences between the groups may therefore reflect factors that were not measured in this study and cannot be separated from the intervention. Although ANCOVA adjusted for baseline scores and BMI (Vickers and Altman, 2001), two features warrant particular caution: academic major differed between groups (*p* = 0.027), and baseline EAT-26 scores were higher in the experimental group (SMD = 0.35). The FAS also showed a baseline SMD of 0.33. The *post hoc* major-adjusted robustness model showed the same pattern across outcomes, but residual confounding by these and other unmeasured factors remains possible. Sample-size planning targeted the primary outcome of body appreciation, and no separate power planning was conducted for the secondary or exploratory outcomes; the analysis of disordered eating risk had limited power to detect small between-group differences, and its non-significant result is not evidence that no difference exists. Because *p*-values were not adjusted across the secondary and exploratory analyses, including the exploratory mediation models, these findings carry an increased risk of Type I error and are interpreted as supporting evidence. They are therefore read with emphasis on effect sizes, confidence intervals, and the consistency of the overall pattern; the sensitivity analyses assessed the robustness of the findings. The study was not prospectively registered, so the findings should be interpreted as preliminary rather than confirmatory. In addition, the comparison condition matched the intervention in duration and scheduling but not novelty, expectancy, instructor attention to embodied experience, or reflective group activities. The observed group differences are therefore relative to regular university physical education electives and do not isolate the efficacy of individual course components. Session-level content, social interaction, and physical activity intensity in the comparison condition were not systematically recorded, so differences in these dimensions between the groups cannot be described.

Additional boundaries concern measurement and generalizability. Post-test non-completion, attention-check exclusions, and per-outcome missingness reduced the effective analytic n despite the study meeting its pre-attrition planning target. The total number of participants per outcome therefore differs across the reported analyses, ranging from 94 to 101 in Table 3 and from 93 to 100 in the main ANCOVA models in Table 4; the exploratory mediation models reported in Section 3.5 use 93 to 98 participants. Outcomes were self-reported and assessed immediately after the intervention; the EAT-26 was used as a screening rather than diagnostic measure; and the conventional threshold of 20 may underestimate elevated risk relative to lower Chinese clinical cutoffs (Kang et al., 2017). The SBC, FAS, and OBCS-BS were Chinese adaptations requiring further validation, and the single-university female undergraduate sample limits generalizability.

### 4.7 Future research

Future studies should employ prospectively registered randomized designs where feasible, incorporate active comparison conditions matched for contact and enjoyment, and add follow-up assessments to test the maintenance of effects. Larger samples would improve power to test indirect pathways and to model embodiment and body surveillance using longitudinal designs that establish temporal precedence. Examining boundary conditions—such as effects among students with elevated baseline disordered eating risk—and integrating behavioral or interoceptive indices would further clarify how, and for whom, somatic dance supports positive body image.

## 5 Conclusion

Relative to regular university physical education electives, the somatic dance course was associated with greater short-term increases in body appreciation, embodied self-awareness, and functionality appreciation and greater decreases in body surveillance among female university students, while group differences in disordered eating risk were limited in this short-term, non-clinical context. Exploratory analyses did not establish embodiment as a mediator. Overall, the findings provide preliminary evidence that a non-aesthetic, embodiment-informed somatic dance course is a feasible addition to campus positive-body-image promotion, and they support its further evaluation in rigorous randomized designs with longer-term follow-up.

## Data Availability

The anonymized raw data supporting the conclusions of this article will be made available by the authors upon reasonable request.

## References

[ref1] Alleva J. M. Tylka T. L. (2021). Body functionality: a review of the literature. Body Image 36, 149–171. doi: 10.1016/j.bodyim.2020.11.006, 33321273

[ref2] Alleva J. M. Tylka T. L. Kroon Van Diest A. M. (2017). The functionality appreciation scale (FAS): development and psychometric evaluation in U.S. community women and men. Body Image 23, 28–44. doi: 10.1016/j.bodyim.2017.07.008, 28822275

[ref3] Anderberg I. Kemps E. Prichard I. (2025). The link between the use of diet and fitness monitoring apps, body image and disordered eating symptomology: a systematic review. Body Image 52:101836. doi: 10.1016/j.bodyim.2024.101836, 39671845

[ref4] Anderson C. Linardon J. (2024). Body image facets as predictors of muscularity-oriented disordered eating in women: findings from a prospective study. Body Image 51:101805. doi: 10.1016/j.bodyim.2024.101805, 39476592

[ref5] Davies A. E. Burnette C. B. Ravyts S. G. Mazzeo S. E. (2022). A randomized control trial of expand your horizon: an intervention for women with weight bias internalization. Body Image 40, 138–145. doi: 10.1016/j.bodyim.2021.12.006, 34954448 PMC8891047

[ref6] Fredrickson B. L. Roberts T.-A. (1997). Objectification theory: toward understanding women's lived experiences and mental health risks. Psychol. Women Q. 21, 173–206. doi: 10.1111/j.1471-6402.1997.tb00108.x

[ref7] Garner D. M. Olmsted M. P. Bohr Y. Garfinkel P. E. (1982). The eating attitudes test: psychometric features and clinical correlates. Psychol. Med. 12, 871–878. doi: 10.1017/S0033291700049163, 6961471

[ref8] Golmohamadi S. Murray K. Farrer L. (2025). Examining the unique benefits of functionality appreciation for body image, self-objectification, and well-being in women. Body Image 53:101936. doi: 10.1016/j.bodyim.2025.101936, 40639269

[ref9] Guo Y. Xiao L. Dang J. Li B. (2025). Self-objectification is (still) gendered: a meta-analysis across measures and societal contexts. Body Image 55:101972. doi: 10.1016/j.bodyim.2025.101972, 41043202

[ref10] Jankauskiene R. Balciuniene V. Rutkauskaite R. Pajaujiene S. Baceviciene M. (2024). The effects of nirvana fitness and functional training on the body appreciation of young women: a non-randomized controlled trial. Front. Psychol. 15:1412259. doi: 10.3389/fpsyg.2024.1412259, 39011281 PMC11247536

[ref11] Kang Q. Chan R. C. K. Li X. Arcelus J. Yue L. Huang J. . (2017). Psychometric properties of the Chinese version of the eating attitudes test in young female patients with eating disorders in mainland China. Eur. Eat. Disord. Rev. 25, 613–617. doi: 10.1002/erv.2560, 28994215

[ref12] Koch S. C. Riege R. F. F. Tisborn K. Biondo J. Martin L. Beelmann A. (2019). Effects of dance movement therapy and dance on health-related psychological outcomes: a meta-analysis update. Front. Psychol. 10:1806. doi: 10.3389/fpsyg.2019.01806, 31481910 PMC6710484

[ref13] Levinson J. A. Kinkel-Ram S. Myers B. Hunger J. M. (2024). A systematic review of weight stigma and disordered eating cognitions and behaviors. Body Image 48:101678. doi: 10.1016/j.bodyim.2023.101678, 38278088 PMC11180546

[ref14] Lewis-Smith H. Garbett K. Chaudhry A. Dhillon M. Shroff H. White P. . (2023). Evaluating a body image school-based intervention in India: a randomized controlled trial. Body Image 44, 148–156. doi: 10.1016/j.bodyim.2022.12.006, 36608434

[ref15] Linardon J. (2021). Positive body image, intuitive eating, and self-compassion protect against the onset of the core symptoms of eating disorders: a prospective study. Int. J. Eat. Disord. 54, 1967–1977. doi: 10.1002/eat.23623, 34599619

[ref16] Linardon J. McClure Z. Tylka T. L. Fuller-Tyszkiewicz M. (2022). Body appreciation and its psychological correlates: a systematic review and meta-analysis. Body Image 42, 287–296. doi: 10.1016/j.bodyim.2022.07.003, 35878528

[ref17] Liu C. Jarman H. K. Messer M. Linardon J. (2025). Predictors of functionality appreciation: prospective findings. Body Image 53:101881. doi: 10.1016/j.bodyim.2025.101881, 40154014

[ref18] Ma J. Wang K. Tylka T. L. (2022). Psychometric properties of a mandarin Chinese version of the body appreciation Scale-2 with residents from Chinese mainland. Body Image 42, 110–119. doi: 10.1016/j.bodyim.2022.05.007, 35691102

[ref19] McKinley N. M. Hyde J. S. (1996). The objectified body consciousness scale: development and validation. Psychol. Women Q. 20, 181–215. doi: 10.1111/j.1471-6402.1996.tb00467.x

[ref20] Messer M. Tylka T. L. Fuller-Tyszkiewicz M. Linardon J. (2022). Does body appreciation predict decreases in eating pathology via intuitive eating? A longitudinal mediation analysis. Body Image 43, 107–111. doi: 10.1016/j.bodyim.2022.08.014, 36113280

[ref21] Modica C. A. (2021). Modeling the associations between internal body orientation, body appreciation, and intuitive eating among early-adult and middle-adult men and women: a multigroup structural invariance analysis. Body Image 39, 1–15. doi: 10.1016/j.bodyim.2021.05.013, 34119807

[ref22] Noll S. M. Fredrickson B. L. (1998). A mediational model linking self-objectification, body shame, and disordered eating. Psychol. Women Q. 22, 623–636. doi: 10.1111/j.1471-6402.1998.tb00181.x

[ref23] Piran N. (2016). Embodied possibilities and disruptions: the emergence of the experience of embodiment construct from qualitative studies with girls and women. Body Image 18, 43–60. doi: 10.1016/j.bodyim.2016.04.007, 27236476

[ref24] Price C. J. Thompson E. A. (2007). Measuring dimensions of body connection: body awareness and bodily dissociation. J. Altern. Complement. Med. 13, 945–953. doi: 10.1089/acm.2007.0537, 18047441 PMC3029599

[ref25] Ramesh S. Glashouwer K. A. Alleva J. M. (2026). Functionality appreciation among adolescents and emerging adults with eating disorders: associations with body appreciation, self-esteem, and eating disorder symptoms. Body Image 56:102056. doi: 10.1016/j.bodyim.2026.102056, 41713164

[ref26] Raspovic A. Prichard I. Salim A. Yager Z. Hart L. M. (2023). Body image profiles combining body shame, body appreciation and body mass index differentiate dietary restraint and exercise amount in women. Body Image 46, 117–122. doi: 10.1016/j.bodyim.2023.05.007, 37290141

[ref27] Saunders J. Nutter S. Waugh R. Hayden K. A. (2024). Testing body-related components of objectification theory: a meta-analysis of the relations between body shame, self-objectification, and body dissatisfaction. Body Image 50:101738. doi: 10.1016/j.bodyim.2024.101738, 38850716

[ref28] Stice E. (2002). Risk and maintenance factors for eating pathology: a meta-analytic review. Psychol. Bull. 128, 825–848. doi: 10.1037/0033-2909.128.5.825, 12206196

[ref29] Stice E. Onipede Z. A. Marti C. N. (2021). A meta-analytic review of trials that tested whether eating disorder prevention programs prevent eating disorder onset. Clin. Psychol. Rev. 87:102046. doi: 10.1016/j.cpr.2021.102046, 34048952

[ref30] Stice E. Shaw H. Burton E. Wade E. (2006). Dissonance and healthy weight eating disorder prevention programs: a randomized efficacy trial. J. Consult. Clin. Psychol. 74, 263–275. doi: 10.1037/0022-006X.74.2.263, 16649871 PMC1479305

[ref31] Toole A. M. LoParo D. Craighead L. W. (2021). Self-compassion and dissonance-based interventions for body image distress in young adult women. Body Image 38, 191–200. doi: 10.1016/j.bodyim.2021.04.001, 33940550

[ref32] Tylka T. L. Wood-Barcalow N. L. (2015a). The body appreciation Scale-2: item refinement and psychometric evaluation. Body Image 12, 53–67. doi: 10.1016/j.bodyim.2014.09.006, 25462882

[ref33] Tylka T. L. Wood-Barcalow N. L. (2015b). What is and what is not positive body image? Conceptual foundations and construct definition. Body Image 14, 118–129. doi: 10.1016/j.bodyim.2015.04.001, 25921657

[ref34] Verzijl C. L. Duan J. Wilfred S. A. Becker C. B. Welch E. Stewart T. M. (2021). Tailoring a dissonance-based body image intervention for adult women in a proof of concept trial: the women's body initiative. Body Image 36, 269–275. doi: 10.1016/j.bodyim.2021.01.001, 33486295 PMC8995137

[ref35] Vickers A. J. Altman D. G. (2001). Analysing controlled trials with baseline and follow up measurements. BMJ 323, 1123–1124. doi: 10.1136/bmj.323.7321.1123, 11701584 PMC1121605

[ref36] Wang H. Alivi M. A. Mustafa S. E. (2025). Unveiling the relationship between social media and self-objectification: a three-level meta-analysis. Body Image 53:101895. doi: 10.1016/j.bodyim.2025.101895, 40311165

[ref37] Wu Y. Kemps E. Prichard I. (2024). Digging into digital buffets: a systematic review of eating-related social media content and its relationship with body image and eating behaviours. Body Image 48:101650. doi: 10.1016/j.bodyim.2023.101650, 38039952

[ref38] Xu R. Chen C. (2025). Sweet words, secretly sour? Ambivalent sexism may predict disordered eating in Chinese women: the roles of self-objectification, body image comparison, and partner intimacy. J. Eat. Disord. 13:241. doi: 10.1186/s40337-025-01425-5, 41152996 PMC12570652

[ref39] Yamamiya Y. Shimai S. Homan K. J. (2021). Exploring the gratitude model of body appreciation and intuitive eating among Japanese women. Body Image 36, 230–237. doi: 10.1016/j.bodyim.2020.12.005, 33387961

[ref40] Yu W. Cui L. Liu C. Wang J. Li J. Lipowski M. (2026). Social media use and disordered eating in young adults: the protective and risk pathways of physical activity and body image. Front. Psychol. 16:1699756. doi: 10.3389/fpsyg.2025.1699756, 41613840 PMC12847240

[ref41] Zhang Y. Xiong S. Yao N. Chen J. (2025). Social media and body image in young women: a meta-analysis of experimental studies on video- versus image-based platforms. Body Image 55:101991. doi: 10.1016/j.bodyim.2025.101991, 41161231

[ref42] Zhao Y. Yin M. Huang M. Chen X. (2025). The effectiveness of randomized controlled psychosocial interventions on body image among nonclinical women: a systematic review and meta-analysis. Body Image 52:101843. doi: 10.1016/j.bodyim.2024.101843, 39756142

[ref43] Zhu H. Wen Y. (2025). Effects of a basketball intervention on female college students' positive body image: chain mediated role of the experience of embodiment and self-compassion. Front. Psychol. 16:1593420. doi: 10.3389/fpsyg.2025.1593420, 40978243 PMC12443768

