# Supplementary material for: Effects of a somatic dance course on body image, embodied self-awareness, and disordered eating risk in female university students: a quasi-experimental study
Source: Front Psychol. 2026 Oct 19;17:1933772. doi: 10.3389/fpsyg.2026.1933772 (PMC13724601; doi:10.3389/fpsyg.2026.1933772)
Supplement: Supplementary file 1 [file Supplementary_file_1.DOCX]

**Supplementary Material 1. Somatic Dance Intervention Manual**

# Program Overview

| **Element** | **Specification** |
| --- | --- |
| Duration | 8 weeks |
| Frequency | Two sessions per week |
| Session length | 60 minutes |
| Total contact time | 960 minutes |
| Setting | University movement studio without mirrors |
| Delivery mode | Group-based, non-performative, non-aesthetic movement sessions |
| Instructor | Certified somatic movement educator trained in the study protocol |
| Core frame | Embodiment-informed movement emphasizing internal sensation, body agency, functionality, and non-judgmental attention |

# Core Delivery Principles

- Use no appearance-, weight-, shape-, or body-size-focused comments.
- Avoid technical ranking, performance correction, or aesthetic evaluation.
- Offer choice in intensity, range, distance, participation, and stopping.
- Direct attention toward internal bodily sensations, breath, effort, support, comfort, agency, and function.
- Normalize different movement capacities and allow seated, standing, or reduced-range alternatives.
- Treat reflective sharing as optional and non-evaluative; participants may pass without explanation.

# Standard Session Structure

| **Component** | **Time** | **Purpose** | **Typical Activities** |
| --- | --- | --- | --- |
| Interoceptive awareness practice | 5 min | Shift attention from external appearance evaluation to internal sensation. | Guided body scan, breath observation, contact with floor, muscle tension/temperature noticing. |
| Non-judgmental movement exploration | 40 min | Practice agency, sensation-led movement, functionality appreciation, and non-evaluative movement. | Improvisation, weight shift, gesture, walking, mirroring with opt-out choices, boundary exploration. |
| Group sharing and body journaling | 15 min | Consolidate bodily awareness and self-care intentions. | Brief optional sharing, written body journal prompts, closing self-care cue. |

# Weekly Themes and Targets

| **Week** | **Theme** | **Theoretical Target** | **Main Activities** |
| --- | --- | --- | --- |
| 1 | Safety and boundaries | Establish psychological and physical safety; introduce non-judgmental body attention. | orientation to safety norms; body scan in standing or seated position; small-range improvisation with choice of distance; journal entry on safety and comfort |
| 2 | Breath and grounding | Strengthen interoceptive awareness through breath, weight, and grounding cues. | breath observation; weight-shift and grounding exploration; slow walking with attention to feet and support; reflection on breath and stability |
| 3 | Non-evaluative movement | Reduce appearance and performance monitoring by exploring movement without right/wrong standards. | movement without mirrors or correction; improvised gestures at varied tempo; noticing evaluation language and returning to sensation; journal entry on non-judgment |
| 4 | Functionality appreciation | Redirect attention toward what the body can sense, support, carry, express, and do. | movement for reach, balance, support, and coordination; pair observation focused on function rather than appearance; gratitude for body functions; reflection on daily bodily capacities |
| 5 | Emotion and bodily cues | Notice links among emotion, body sensation, movement impulse, and self-care needs. | mapping emotion to posture, breath, and movement impulse; movement phrase for release or containment; choice-based sharing; self-care intention |
| 6 | Body boundaries and agency | Practice choosing distance, intensity, direction, and consent in movement. | personal-space exploration; yes/no movement cues; choosing intensity and stopping points; reflection on bodily boundaries |
| 7 | Relational embodiment | Explore connection with others while maintaining bodily agency and boundaries. | parallel movement; mirroring with opt-out choices; group rhythm without performance ranking; reflection on connection and autonomy |
| 8 | Integration and self-care | Consolidate preferred practices and translate body-affirming attention into daily self-care. | review of preferred practices; integrated improvisation; future self-care planning; closing reflection |

# Risk Management and Participant Support

- Participants were reminded that they could pause, sit, observe, or leave an activity if discomfort emerged.
- The instructor redirected appearance- or weight-focused comments toward body sensation, comfort, function, or self-care.
- Any acute distress, injury, or request for support was documented and referred according to the study's adverse-event procedure.
- The intervention was not presented as treatment for eating disorders or psychological crisis; students requiring specialized care were directed to appropriate services.

# Materials

Open studio space, optional mats or chairs, music selected for steady rhythm rather than performance display, printed or electronic body-journal prompts, and instructor fidelity checklist.
