# Supplementary material for: Effects of a somatic dance course on body image, embodied self-awareness, and disordered eating risk in female university students: a quasi-experimental study
Source: Front Psychol. 2026 Oct 19;17:1933772. doi: 10.3389/fpsyg.2026.1933772 (PMC13724601; doi:10.3389/fpsyg.2026.1933772)
Supplement: Supplementary file 2 [file Supplementary_file_2.DOCX]

**Supplementary Material 2. Instructor Scripts and Body Journal Prompts**

# General Opening Script

Before we begin, notice that this session is not about how movement looks. There is no correct shape to copy and no performance standard to meet. You may choose a smaller movement, a slower rhythm, a seated option, or a pause at any time. Let attention move toward breath, contact with the floor, muscle sensation, effort, comfort, and what your body is telling you today.

# General Redirection Script

If thoughts about appearance, weight, or comparison arise, acknowledge them without blame and return attention to sensation, support, breath, range, function, or choice. The purpose is to notice the body from the inside rather than to evaluate it from the outside.

# Week-Specific Scripts and Journal Prompts

## Week 1. Safety and boundaries

Theoretical target: Establish psychological and physical safety; introduce non-judgmental body attention.

Core instructor cue: Notice what feels safe enough today. You may make any movement smaller, slower, or stop at any time.

Movement focus: orientation to safety norms; body scan in standing or seated position; small-range improvisation with choice of distance; journal entry on safety and comfort.

Body journal prompts:

- Where did my body feel safest today?
- What helped me feel more comfortable or less comfortable?
- What boundary do I want to respect this week?

## Week 2. Breath and grounding

Theoretical target: Strengthen interoceptive awareness through breath, weight, and grounding cues.

Core instructor cue: Let attention rest on breath, weight, and contact with the floor. There is no correct posture to achieve.

Movement focus: breath observation; weight-shift and grounding exploration; slow walking with attention to feet and support; reflection on breath and stability.

Body journal prompts:

- What did I notice about breath, weight, or grounding?
- Did any sensation change during the session?
- How could I use one grounding cue outside class?

## Week 3. Non-evaluative movement

Theoretical target: Reduce appearance and performance monitoring by exploring movement without right/wrong standards.

Core instructor cue: If an evaluative thought appears, acknowledge it and return to sensation, effort, rhythm, or comfort.

Movement focus: movement without mirrors or correction; improvised gestures at varied tempo; noticing evaluation language and returning to sensation; journal entry on non-judgment.

Body journal prompts:

- When did I notice self-evaluation?
- What helped me return to bodily sensation?
- What would non-judgmental movement look like in daily life?

## Week 4. Functionality appreciation

Theoretical target: Redirect attention toward what the body can sense, support, carry, express, and do.

Core instructor cue: Explore what your body can do for you: support, carry, reach, breathe, protect, communicate, and rest.

Movement focus: movement for reach, balance, support, and coordination; pair observation focused on function rather than appearance; gratitude for body functions; reflection on daily bodily capacities.

Body journal prompts:

- What body function did I appreciate today?
- How did my body support me during movement?
- What is one non-appearance-based reason to care for my body?

## Week 5. Emotion and bodily cues

Theoretical target: Notice links among emotion, body sensation, movement impulse, and self-care needs.

Core instructor cue: Let movement be a way to notice emotion rather than to display it. Choose the amount of expression that feels workable.

Movement focus: mapping emotion to posture, breath, and movement impulse; movement phrase for release or containment; choice-based sharing; self-care intention.

Body journal prompts:

- What emotion was easiest or hardest to notice in the body?
- What body cue might signal a need for rest, support, or expression?
- What self-care action follows from that cue?

## Week 6. Body boundaries and agency

Theoretical target: Practice choosing distance, intensity, direction, and consent in movement.

Core instructor cue: Practice choosing distance, direction, and intensity. A boundary can be adjusted at any moment.

Movement focus: personal-space exploration; yes/no movement cues; choosing intensity and stopping points; reflection on bodily boundaries.

Body journal prompts:

- When did I choose distance, intensity, or stopping?
- What did my body tell me about boundaries?
- Where can I practice a clearer boundary this week?

## Week 7. Relational embodiment

Theoretical target: Explore connection with others while maintaining bodily agency and boundaries.

Core instructor cue: Stay connected to your own sensations while noticing the group. Participation does not require imitation or performance.

Movement focus: parallel movement; mirroring with opt-out choices; group rhythm without performance ranking; reflection on connection and autonomy.

Body journal prompts:

- How did I experience connection while staying aware of myself?
- What helped me avoid comparison?
- What kind of group support felt useful?

## Week 8. Integration and self-care

Theoretical target: Consolidate preferred practices and translate body-affirming attention into daily self-care.

Core instructor cue: Select the practices you want to keep using. Let the closing movement be a reminder of care rather than evaluation.

Movement focus: review of preferred practices; integrated improvisation; future self-care planning; closing reflection.

Body journal prompts:

- Which practice do I want to continue?
- What changed in how I attend to my body?
- What is one realistic body-care intention for the next month?

# Closing Script

Take a moment to notice one sensation, one function, or one boundary that you want to carry with you after class. The aim is not to fix the body or judge the body, but to stay in a more respectful relationship with it.
