# Supplementary material for: Effects of a somatic dance course on body image, embodied self-awareness, and disordered eating risk in female university students: a quasi-experimental study
Source: Front Psychol. 2026 Oct 19;17:1933772. doi: 10.3389/fpsyg.2026.1933772 (PMC13724601; doi:10.3389/fpsyg.2026.1933772)
Supplement: Supplementary file 3 [file Supplementary_file_3.DOCX]

**Supplementary Material 3. Intervention Fidelity Checklist**

# Checklist Items

| **Item** | **Response Format** | **Operational Definition** |
| --- | --- | --- |
| Interoceptive awareness practice completed | Yes/No | Guided attention to breath, body sensation, contact, tension, or temperature. |
| Non-judgmental movement exploration completed | Yes/No | Improvisational movement without appearance, weight, or technical ranking emphasis. |
| Group sharing/body journal completed | Yes/No | Optional reflective sharing and written prompt completion. |
| Session script followed | Yes/No | Core theme and wording delivered as planned. |
| Appearance-evaluation language observed | Yes/No | Any appearance, shape, or attractiveness evaluation by instructor or group requiring redirection. |
| Weight-loss/body-shaping language observed | Yes/No | Any classroom statement referring to weight, body shape, or weight-related body-shaping concerns, including a student-initiated mention of weight; general appearance-evaluation language is coded separately. |
| Technical ranking/performance evaluation observed | Yes/No | Any technical ranking, movement-performance evaluation, or similar evaluative feedback. |
| Protocol deviation observed | Yes/No | A deviation affecting a prespecified course setting, module, or teaching process. A background-music substitution that does not affect course goals, core modules, duration, or teaching process is recorded separately and is not counted here. |
| Observer note | Text | Brief explanation of deviations, redirections, or contextual events. |
| Temporary content substitution | Yes/No | A temporary instructor content substitution that does not affect course goals, core modules, duration, or teaching process. |

# Session-Level Fidelity Summary

| **Session** | **Date** | **Week** | **Theme** | **Interoceptive** | **Non-judgmental** | **Sharing/journal** | **Script** | **Appearance evaluation** | **Weight/body-shaping** | **Technical ranking** | **Protocol deviation** | **Observer-rated score** | **Observer note** | **Temporary content substitution** |
| --- | --- | --- | --- | --- | --- | --- | --- | --- | --- | --- | --- | --- | --- | --- |
| SD-S01 | 2025-09-15 | 1 | Safety and boundaries | 1 | 1 | 1 | 1 | 0 | 1 | 0 | 0 | 96 | A student mentioned weight; the instructor redirected discussion to bodily sensation. | 0 |
| SD-S02 | 2025-09-18 | 1 | Safety and boundaries | 1 | 1 | 0 | 0 | 1 | 0 | 0 | 1 | 62 | A brief appearance-related comment occurred during sharing; the instructor redirected the group to internal bodily sensation. The planned sharing/journal segment was shortened because of time overrun; no safety concern was observed. | 0 |
| SD-S03 | 2025-09-22 | 2 | Breath and grounding | 1 | 1 | 1 | 1 | 0 | 0 | 0 | 1 | 100 | Temporary room adjustment. | 0 |
| SD-S04 | 2025-09-25 | 2 | Breath and grounding | 1 | 1 | 1 | 1 | 0 | 0 | 0 | 0 | 100 |  | 0 |
| SD-S05 | 2025-09-29 | 3 | Non-evaluative movement | 1 | 1 | 1 | 1 | 0 | 0 | 0 | 0 | 99 |  | 0 |
| SD-S06 | 2025-09-30 | 3 | Non-evaluative movement | 1 | 1 | 1 | 1 | 0 | 1 | 0 | 0 | 98 | A student mentioned weight; the instructor redirected discussion to bodily sensation. | 0 |
| SD-S07 | 2025-10-09 | 4 | Functionality appreciation | 1 | 1 | 1 | 1 | 0 | 0 | 0 | 0 | 96 |  | 0 |
| SD-S08 | 2025-10-10 | 4 | Functionality appreciation | 1 | 1 | 0 | 1 | 0 | 0 | 0 | 1 | 77 | Temporary room adjustment; the group-sharing/body-journal segment was not completed. | 0 |
| SD-S09 | 2025-10-13 | 5 | Emotion and bodily cues | 1 | 1 | 1 | 1 | 0 | 0 | 0 | 0 | 100 |  | 0 |
| SD-S10 | 2025-10-16 | 5 | Emotion and bodily cues | 1 | 1 | 1 | 1 | 0 | 0 | 0 | 0 | 99 |  | 0 |
| SD-S11 | 2025-10-20 | 6 | Body boundaries and agency | 1 | 1 | 1 | 1 | 0 | 0 | 0 | 1 | 88 | Minor timing deviation due to room transition; all core embodiment and non-judgmental components were completed. No safety concern was observed. | 0 |
| SD-S12 | 2025-10-23 | 6 | Body boundaries and agency | 1 | 1 | 1 | 1 | 0 | 0 | 0 | 0 | 84 | The instructor changed one music segment. | 1 |
| SD-S13 | 2025-10-27 | 7 | Relational embodiment | 1 | 1 | 0 | 0 | 0 | 0 | 0 | 1 | 82 | Partner/group activity ran over time, so the closing sharing/journal and full script were shortened. Core movement principles were retained; no safety concern was observed. | 0 |
| SD-S14 | 2025-10-30 | 7 | Relational embodiment | 1 | 1 | 1 | 1 | 0 | 0 | 0 | 1 | 100 | Temporary room adjustment. | 0 |
| SD-S15 | 2025-11-03 | 8 | Integration and self-care | 1 | 1 | 1 | 1 | 0 | 0 | 0 | 0 | 96 | The instructor changed one music segment. | 1 |
| SD-S16 | 2025-11-06 | 8 | Integration and self-care | 1 | 1 | 0 | 1 | 0 | 0 | 0 | 1 | 84 | Final reflection discussion exceeded the planned time, so written journaling was shortened. No safety concern was observed. | 0 |

# Scoring Summary

Session-level fidelity scores were observer-rated global scores ranging from 0 to 100. The score was recorded separately from the four binary completion indicators and reflected the observer's overall assessment of protocol adherence, including completion of core components, observed deviations, and field notes. The ratings were made by two recorders who were not involved in delivering the course, working in a primary/backup arrangement, and session records were usually agreed through discussion. The mean fidelity score was calculated as the arithmetic mean of the 16 raw session-level scores: 91.3 out of 100 (range 62-100). A score of 100 indicates that all core components were delivered as planned; minor setting deviations that did not affect core components, such as a temporary room adjustment, were recorded but did not necessarily reduce the score.
