# Supplementary material for: Effects of a somatic dance course on body image, embodied self-awareness, and disordered eating risk in female university students: a quasi-experimental study
Source: Front Psychol. 2026 Oct 19;17:1933772. doi: 10.3389/fpsyg.2026.1933772 (PMC13724601; doi:10.3389/fpsyg.2026.1933772)
Supplement: Supplementary file 4 [file Table_1.docx]

**Supplementary Table S1. Psychometric Checks for Chinese Versions and Adaptations Used in the Present Study**

| **Scale** | **Time point** | **Items** | **Cronbach's alpha** | **McDonald's omega** | **Corrected item-total r** | **CFA model** | **CFI** | **TLI** | **RMSEA** | **SRMR** | **Loading range** |
| --- | --- | --- | --- | --- | --- | --- | --- | --- | --- | --- | --- |
| BAS-2 | Pretest | 10 | 0.880 | 0.887 | 0.42-0.66 | One-factor model | 0.956 | 0.944 | 0.058 | 0.044 | 0.50-0.79 |
| BAS-2 | Posttest | 10 | 0.900 | 0.906 | 0.48-0.71 | One-factor model | 0.964 | 0.954 | 0.052 | 0.039 | 0.54-0.82 |
| SBC | Pretest | 20 | 0.820 | 0.833 | 0.31-0.58 | Two-factor correlated model | 0.932 | 0.921 | 0.064 | 0.057 | 0.39-0.72 |
| SBC | Posttest | 20 | 0.860 | 0.871 | 0.38-0.63 | Two-factor correlated model | 0.946 | 0.936 | 0.057 | 0.050 | 0.44-0.76 |
| EAT-26 | Pretest | 26 | 0.850 | 0.862 | 0.29-0.61 | Three-factor model | 0.912 | 0.901 | 0.068 | 0.061 | 0.35-0.74 |
| EAT-26 | Posttest | 26 | 0.830 | 0.845 | 0.27-0.59 | Three-factor model | 0.905 | 0.892 | 0.071 | 0.064 | 0.32-0.72 |
| FAS | Pretest | 7 | 0.870 | 0.879 | 0.49-0.70 | One-factor model | 0.938 | 0.907 | 0.073 | 0.052 | 0.55-0.80 |
| FAS | Posttest | 7 | 0.890 | 0.898 | 0.53-0.73 | One-factor model | 0.961 | 0.941 | 0.059 | 0.041 | 0.58-0.83 |
| OBCS-BS | Pretest | 8 | 0.790 | 0.804 | 0.35-0.58 | One-factor model | 0.951 | 0.932 | 0.066 | 0.047 | 0.43-0.72 |
| OBCS-BS | Posttest | 8 | 0.840 | 0.852 | 0.42-0.65 | One-factor model | 0.968 | 0.956 | 0.054 | 0.036 | 0.49-0.77 |

Note. These analyses provide exploratory evidence on the measurement performance of the scales in the present sample. Confirmatory factor analysis was performed in R 4.3.3 with lavaan. No item-level mean imputation was used for CFA; participants who completed at least 80% of a scale's items were retained for that scale's analysis, and the ordered categorical items were analyzed using the WLSMV estimator with theta parameterization. Scales: Body Appreciation Scale-2 (BAS-2); Scale of Body Connection (SBC); Eating Attitudes Test-26 (EAT-26); Functionality Appreciation Scale (FAS); Objectified Body Consciousness Scale–Body Surveillance subscale (OBCS-BS). For SBC, the correlated two-factor model reflects the original body awareness and bodily dissociation structure; the total mean score was used after reverse-coding dissociation items so that higher scores indicated stronger body connection. CFA, confirmatory factor analysis; CFI, comparative fit index; TLI, Tucker-Lewis index; RMSEA, root mean square error of approximation; SRMR, standardized root mean square residual.
